# Data on the mechanisms underlying succinate-induced aortic contraction

**DOI:** 10.1016/j.dib.2016.08.022

**Published:** 2016-08-31

**Authors:** Natália A. Gonzaga, Janaina A. Simplicio, Letícia N. Leite, Gabriel T. Vale, José M. Carballido, José C. Alves-Filho, Carlos R. Tirapelli

**Affiliations:** a Departamento de Farmacologia, Faculdade de Medicina de Ribeirão Preto, Universidade de São Paulo (USP), Ribeirão Preto, SP, Brazil; b Laboratório de Farmacologia, DEPCH, Escola de Enfermagem de Ribeirão Preto, USP, Ribeirão Preto, SP, Brazil; c Novartis Institutes for Biomedical Research, Autoimmunity, Transplantation and Inflammation, Basel CH-4002, Switzerland

**Keywords:** Succinate, Contraction, Aorta

## Abstract

We describe the mechanisms underlying the vascular contraction induced by succinate. The data presented here are related to the article entitled “Pharmacological characterization of the mechanisms underlying the vascular effects of succinate” (L.N. Leite, N.A. Gonzaga, J.A. Simplicio, G.T. Vale, J.M. Carballido, J.C. Alves-Filho, C.R. Tirapelli, 2016) [1]. Succinate acts as a signaling molecule by binding to a G-protein-coupled receptor termed GPR91, “Citric acid cycle intermediates as ligands for orphan G-protein-coupled receptors” (W. He, F.J. Miao, D.C. Lin, R.T. Schwandner, Z. Wang, J. Gao, J.L. Chen, H. Tian, L. Ling, 2004) [2]. Here we include data on the contractile effect of succinate in the aorta. Succinate contracted both endothelium-intact and endothelium-denuded aortic rings isolated from male Wistar rats or C57BL/6 mice. Succinate was less effective at inducing contraction in arteries isolated from GPR91-deficient mice, when compared to its vascular effect in aortas from wild type mice. SB203508 (p38MAK inhibitor), SP600125 (JNK inhibitor) and Y27632 (Rho-kinase inhibitor) reduced succinate-induced contraction in both endothelium-intact and endothelium-denuded rat aortic rings, while PD98059 (ERK1/2 inhibitor) did not affect succinate-induced contraction. The contractile response induced by succinate on endothelium-intact and endothelium-denuded rat aortic rings was reduced by indomethacin (non-selective cyclooxygenase inhibitor), H7 (protein kinase C inhibitor), verapamil (Ca^2+^ channel blocker) and tiron (superoxide anion scavenger).

**Specifications Table**

**Table:** 

Subject area	Biology
More specific subject area	Vascular Pharmacology
Type of data	Graph
How data was acquired	Isometric force transducer (TRI201; Panlab, Spain)
Data format	Analyzed
Experimental factors	The thoracic aorta was isolated from male Wistar rats or C57BL/6 mice, cut into rings (4 mm in length) and maintained in organ chambers
Experimental features	Inhibitors of distinct pathways were used to determine the mechanisms underlying succninate-induced contraction
Data source location	Ribeirao Preto, Brazil
Data accessibility	Data are within this article

**Value of the data**

• Succinate displays a direct contractile effect on the vasculature.
• The contraction induced by succinate is partially mediated by GPR91 receptors.
• The data provide insights into the mechanisms underlying the contractile effect of succinate.

## 1 Data

**Fig. 1:**
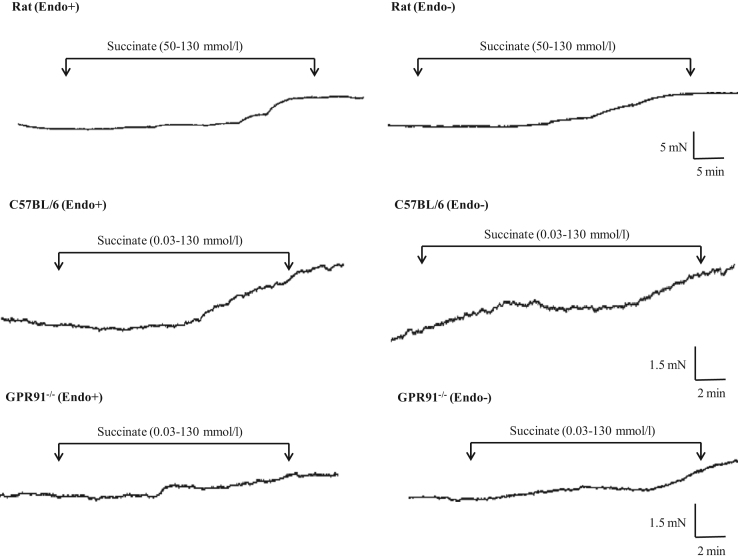
Original traces showing the contractile effect of succinate. The contraction induced by succinate was obtained in endothelium-intact (Endo+) and endothelium-denuded (Endo−) aortic rings isolated from Wistar rats and C57BL/6 or GPR91^−/−^ mice.

**Fig. 2:**
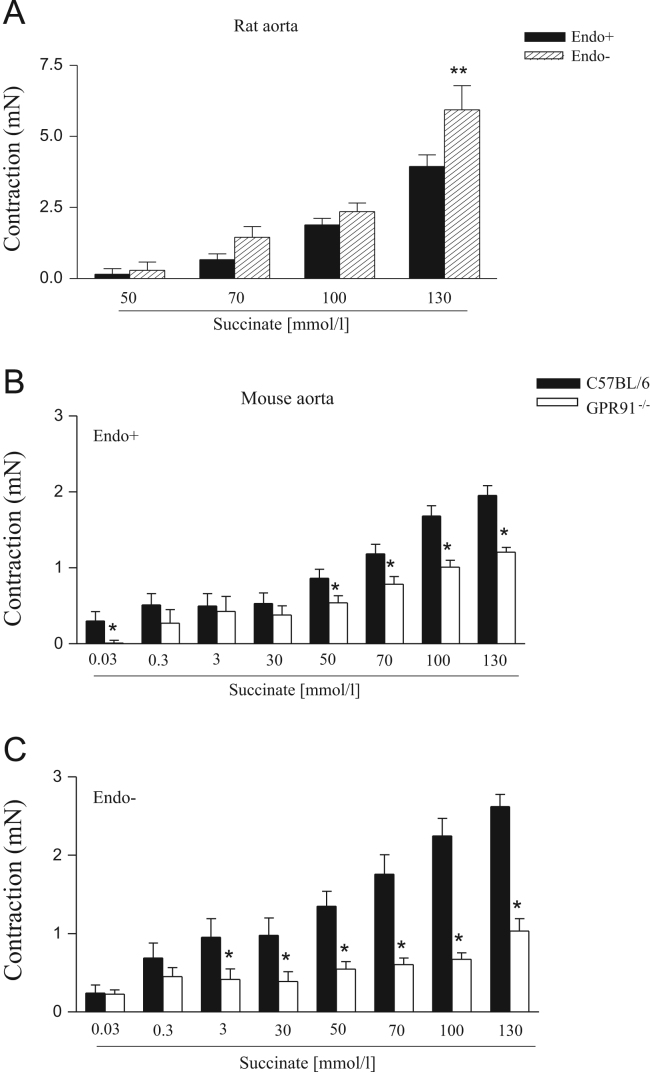
Contractile responses induced by succinate on rat and mouse aortic rings. The contraction induced by succinate was studied on endothelium-intact (Endo+) and endothelium-denuded (Endo−) aortic rings from Wistar rats (A) and C57BL/6 or GPR91^−/−^ mice (B and C). Results are presented as means±S.E.M. of 7 to 9 experiments. *Compared to C57BL/6;**Compared to Endo+ (*P*<0.05, ANOVA followed by Newman–Keuls multiple comparison test).

Data describes the contractile action of succninate. Fig. 1 shows representative traces of the contractile effect of succinate on endothelium-intact and endothelium-denuded aortic rings from Wistar rats and C57BL/6 or GPR91^−/−^ mice. The maximal contraction induced by succinate in endothelium-intact and endothelium-denuded aortas isolated from Wistar rats and C57BL/6 or GPR91-deficient mice are represented on Fig. 2.

**Fig. 3:**
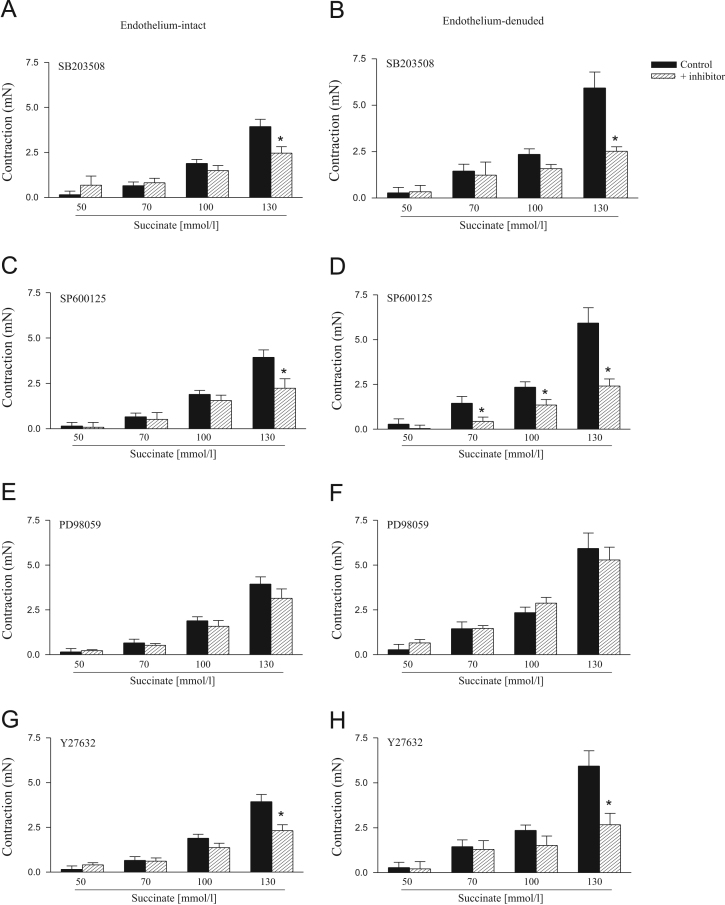
Effect of SB203508, SP600125, PD98059 and Y27632 on succinate-induced contractile response in endothelium-intact (left) and endothelium-denuded (right) rat aortic rings. The contractile response induced by succinate was determined before (control) or after incubation for 30 min with SB203508 (10 µmol/L), SP600125 (10 µmol/L), PD98059 (10 µmol/L) or Y27632 (10 µmol/L). Results are presented as means±S.E.M. of 6 to 10 experiments. *Compared to respective control group (*P*<0.05, ANOVA followed by Newman–Keuls multiple comparison test).

**Fig. 4:**
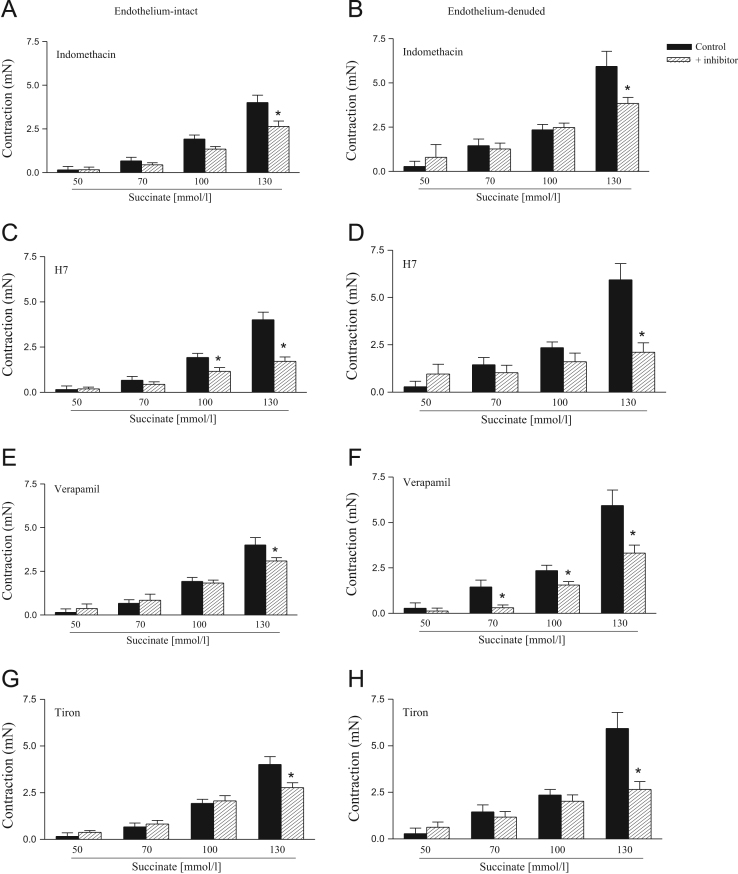
Effect of indomethacin, H7, tiron and verapamil on succinate-induced contractile response in endothelium-intact and denuded rat aortic rings. The contractile response induced by succinate was determined before (control) or after incubation for 30 min with indomethacin (10 µmol/L), H7 (10 µmol/L), verapamil (1 µmol/L) or tiron (100 µmol/L). Results are presented as means±S.E.M. of 6 to 10 experiments. *Compared to respective control group (*P*<0.05, ANOVA followed by Newman–Keuls multiple comparison test).

The effects of SB203508, SP600125, PD98059 and Y27632 on succinate-induced contraction are represented on Fig. 3. The contractile response induced by succinate on endothelium-intact and endothelium-denuded rat aortic rings was reduced by indomethacin, H7, verapamil and tiron (Fig. 4).

## 2 Experimental design, materials and methods

Male Wistar rats (230–250 g) and C57BL/6 or GPR91^−/−^ mice (20–25 g) were anaesthetized intraperitoneally with urethane at 1.25 g/kg (Sigma-Aldrich, St. Louis, MO, USA) and the thoracic aorta was mounted in organ chambers [1]. The contraction induced by succinate (50–130 mmol/L) was evaluated in endothelium-intact and endothelium-denuded rat aortic rings. In another set of experiments, the contractile effect of succinate (0.03–130 mmol/L) was evaluated in endothelium-intact and endothelium-denuded aortic rings from C57BL/6 and GPR91^−/−^ mice [2].

In another set of experiments, the contractile response induced by succinate was evaluated in endothelium-intact or endothelium-denuded rat aortic rings incubated for 30 min with one of the following drugs: SB203508 (10 µmol/L, p38MAK inhibitor), SP600125 (10 µmol/L, JNK inhibitor), PD98059 (10 µmol/L, ERK1/2 inhibitor), Y27632 (10 µmol/L, Rho-kinase inhibitor), H7 (10 μmol/L, protein kinase C inhibitor), indomethacin (10 μmol/L, non-selective cyclooxygenase inhibitor), tiron (superoxide anion scavenger, 100 μmol/L) or verapamil (1 μmol/L, Ca^2+^ channel blocker).

Results are expressed as means±standard errors of the mean (S.E.M.). Statistical analysis was performed using one-way analysis of variance (ANOVA) followed by Newman–Keuls multiple comparison test. *P* values of less than 0.05 were considered significant.
