# Supplementary material for: Data on the mechanisms underlying succinate-induced aortic contraction
Source: Data Brief. 2016 Aug 31;9:206–12. doi: 10.1016/j.dib.2016.08.022 (PMC5021798; doi:10.1016/j.dib.2016.08.022)
Supplement: Supplementary file 1 — Supplementary material [file mmc1.doc]

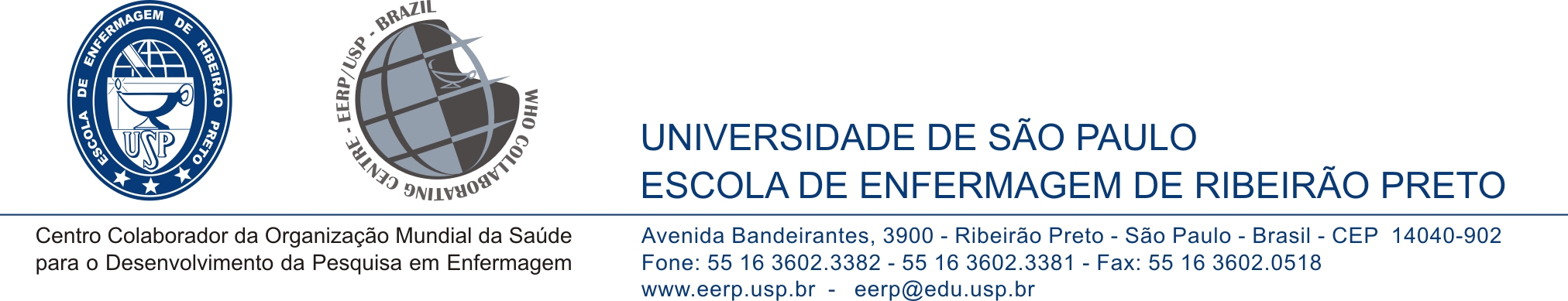


**DIB-D-16-00601**

Title: "Data on the mechanisms underlying succinate-induced aortic contraction".

We declare no conflict of interest.


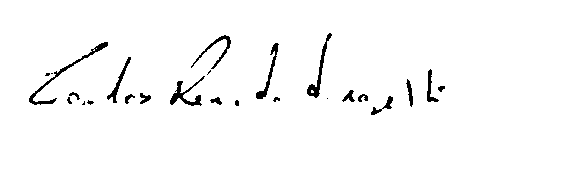


Prof. Carlos R. Tirapelli

Universidade de São Paulo

Avenida Bandeirantes, 3900

14040 902 - Ribeirão Preto, Brazil

crtirapelli@eerp.usp.br
